# Adolescent depression, childhood maltreatment, and the immune system; a role for epigenetic aging?

**DOI:** 10.1016/j.bbih.2025.101080

**Published:** 2025-07-28

**Authors:** Marieke S. Tollenaar, Nicole Creasey, Mirjam C.M. Wever, Karen Knipping, Johan Garssen, Lisanne E.A.M. van Houtum, Wilma G.M. Wentholt, Loes Janssen, Elad Lax, Bernet M. Elzinga

**Affiliations:** a Leiden University, Institute of Psychology and Leiden Institute for Brain and Cognition, the Netherlands; b Faculty of Education, PEDAL Research Centre, University of Cambridge, Cambridge, UK; c Utrecht Institute for Pharmaceutical Sciences, Division Pharmacology, Utrecht University, the Netherlands; d Danone Nutricia Research, Utrecht, the Netherlands; e Department of Child and Adolescent Psychiatry/Psychology, Erasmus MC, University Medical Centre–Sophia, Rotterdam, the Netherlands; f Ariel University, Department of Molecular Biology, Israel

**Keywords:** Depression, Inflammation, Epigenetic aging, Childhood maltreatment, Immune system

## Abstract

**Background:**

Childhood maltreatment is a major risk factor for the development of depression, as well as for imbalances in the immune system, including chronic low-grade inflammation. Less is known about potential immune imbalances in adolescent depression and the role of childhood maltreatment. Furthermore, accelerated epigenetic aging may contribute to the development of inflammation, but has never been examined in the context of adolescent depression.

**Methods:**

We investigated childhood maltreatment, inflammation, and epigenetic aging in 78 healthy adolescents and 33 adolescents with a clinically diagnosed depressive disorder. Childhood maltreatment was assessed via self-report, and inflammatory markers (CRP, IL-1β, IL-6, IL-8, sIgA, and TNF-α) were analyzed in saliva. DNA methylation was analyzed in a subsample (*n* = 48) of both healthy and unmedicated, depressed adolescents to estimate epigenetic age (Horvath and PedBE clocks).

**Results:**

The MANOVA showed that depression was significantly associated with the immune markers, but not with epigenetic aging. Post-hoc tests suggested however that IL-8 levels were reduced, while none of the other markers were affected. Unexpectedly, childhood (emotional) maltreatment was significantly associated with epigenetic age *de*celeration on the PedBE clock (*p* = .01), and not with inflammation. The inflammatory markers were not associated with either of the epigenetic age clocks.

**Conclusions:**

Our results contrast with previous literature in adults, indicating that epigenetic age *ac*celeration and inflammation are not unequivocal indicators of depression and childhood trauma in adolescents. Future studies with larger, more heterogeneous samples should investigate under which circumstances increased or decreased levels of these biological markers indicate vulnerability for health-related outcomes. Better understanding of these mechanisms in adolescence may help to develop appropriate interventions supporting a healthy development of children who experienced maltreatment or depression.

## 1 Introduction

Depression is a debilitating mental health disorder that significantly impacts individuals' psychological well-being and social functioning as well as their physical health outcomes (Clayborne et al., 2019; Friedrich, 2017). Research indicates that the onset of depression often occurs during adolescence, a crucial developmental period, which can lead to worse long-term prognosis and increased vulnerability to recurrence (Copeland et al., 2021; Zisook et al., 2007). In adulthood, depression has been associated with autoimmune and metabolic diseases, including diabetes and cardiovascular diseases (Correll et al., 2017; Danese et al., 2009; Wolkowitz et al., 2011). It is thought that these somatic health issues may be due to chronic low-grade inflammation, which has often been found in individuals with depression (Dantzer, 2012; Lamers et al., 2019; Lindqvist et al., 2017; Pariante, 2017). While chronic inflammation may arise because of the stress and hormonal imbalances associated with depression, dysregulation of the immune system in depression may in part be caused by early-life stressors (Giollabhui et al., 2020; Ravi et al., 2021; Slavich and Irwin, 2014). That is, early adverse experiences like childhood maltreatment may lead to long-lasting changes in immune functioning, which increases susceptibility to both depression and inflammatory diseases (Baldwin and Danese, 2019; Baumeister et al., 2016; Hughes et al., 2017; Lippard and Nemeroff, 2020).

The immune imbalances associated with depression may already begin to emerge during adolescence, with increases in interleukin (IL)-6 and C-Reactive Protein (CRP) being most robust indicators of depressive symptoms, although meta-analytic studies show large differences in outcomes and effects sizes between studies and immune measures (Colasanto et al., 2020; D'Acunto et al., 2019; Toenders et al., 2021). Furthermore, these imbalances may be dependent on the amount of childhood adversities experienced, with strongest immune changes in those with both early life stress and depression (Miller and Cole, 2012; Zajkowska et al., 2021). To develop a comprehensive understanding of early onset depression and its associated health risks, it is important to further investigate the interplay between immune dysregulation and adolescent depression alongside the role of early life stressors in these associations. Hence, the first goal of our study was to examine inflammatory immune markers in a sample of adolescents with depressive disorder in comparison with a matched healthy control group, and to examine whether experiences of childhood maltreatment could explain potential dysregulations. We explored the roles of both emotional and physical maltreatment, as emotional maltreatment in particular has been associated with the development of depression (Nelson et al., 2017). We assessed the immune markers in saliva, which is a less invasive method than blood testing and can be more easily scaled and included in clinical practice (Lim et al., 2016; Shields et al., 2019; Szabo and Slavish, 2021). Using such a less invasive measure may especially be relevant in younger and vulnerable populations, to reduce potential stress and acute immunological responses associated with blood drawing (Weckesser et al., 2014).

Secondly, accelerated biological aging has been implicated as an important factor in the development of immune dysregulations and associated health outcomes in adulthood (Belsky et al., 2017; Franceschi et al., 2018; Levine et al., 2018a; Morrison et al., 2019). Accelerated biological aging occurs when physiological development of the body and cellular senescence progresses at a faster rate than would be expected based on chronological age and may confer increased risk for age-related diseases. One important measure of biological aging is epigenetic aging (Horvath and Raj, 2018), which tracks changes in gene regulation and expression over time via changes in Deoxyribonucleic Acid (DNA) methylation. Research has demonstrated that both childhood trauma and depression are associated with alterations in DNA methylation (Chen et al., 2017; Houtepen et al., 2016; Peng et al., 2018), including those that are implicated in accelerated epigenetic aging (Han et al., 2018; Harvanek et al., 2023; Shindo et al., 2023). These epigenetic changes may help explain some of the immune imbalances observed in individuals with depression (Franceschi et al., 2018; Milaneschi et al., 2020; Shindo et al., 2023). Furthermore, epigenetic age acceleration (EAA) has been found to predict adverse health outcomes and increased mortality risk in adulthood (Chen et al., 2016; Cribb et al., 2022; Levine et al., 2018b), highlighting the significance of understanding its role in the associations between depression, early life stress and the immune system in adolescence.

There have been quite some studies showing associations between early life adversity and accelerated epigenetic aging in children and adolescents (Copeland et al., 2022; Jovanovic et al., 2017; Marini et al., 2020; Sumner et al., 2019, 2023a; Tang et al., 2020). With regards to EAA in adolescent depression, no studies to date have included adolescents with a clinically diagnosed depression. So far, some studies have shown associations of EAA with internalizing symptoms (Caro et al., 2023; Suarez et al., 2018a; Tollenaar et al., 2019) and others with depressive symptoms in childhood and adolescence (Perret et al., 2023), although not all studies consistently found accelerated aging (Caro et al., 2023; Ingram et al., 2024). Associations of EAA with depressive symptoms may moreover be dependent on experiences of early life stress (Dammering et al., 2021; Sumner et al., 2019; Zhang et al., 2023). Furthermore, to our knowledge, only one study has previously examined associations between parent-reported internalizing symptoms, an inflammatory marker, and epigenetic aging in children (Raffington et al., 2024), but not how inflammation and epigenetic aging may interrelate in adolescents with a clinical diagnosis of depression. Hence, as a second aim, we examined whether adolescents with depression show accelerated epigenetic age compared to those without depression, and whether accelerated epigenetic aging was related to salivary markers of inflammation. We also examined whether childhood maltreatment may explain associations between depression and EAA. Several epigenetic clocks exist to assess EAA, we therefore included both the multi-tissue clock developed by Horvath (2013) and the Pediatric-Buccal-Epigenetic clock (PedBE; Mcewen et al., 2019) that was specifically trained on buccal cells in children.

In summary, we first studied the extent to which adolescent depression is associated with inflammation, as assessed by the levels of IL-1β, IL-6, IL-8, Tumor Necrosis Factor α (TNF-α), Secretory Immunoglobulin A (sIgA) and CRP in saliva, and whether this association can be attributed to early life stress, as assessed by experiences of childhood maltreatment. Secondly, we examined the role of epigenetic aging in these associations. We expected that adolescents with depression would show higher levels of inflammation and a higher rate of epigenetic aging than adolescents without depression. Furthermore, we expected that higher levels of experienced childhood maltreatment in the adolescents with depression may explain some of these associations. Lastly, we explored whether emotional versus physical maltreatment contributes more strongly to any explained variance. Our hypotheses regarding EAA were included in the preregistration of a larger study project (see https://osf.io/8k4ga: Secondary analyses b).

## 2 Methods

### 2.1 Participants

Participants for this study were drawn from the RE-PAIR study (Janssen et al., 2024; van Houtum et al., 2023; Wever et al., 2024), of which the main objective was to examine parent-adolescent interactions in relation to mood. All adolescents included in this study were between 11 and 17 years old at the time of enrollment, willing to participate, and living with at least one primary caregiver. They were also required to be attending or have completed high school. Both the adolescents and their parent(s) needed to demonstrate a sufficient proficiency in the Dutch language. Adolescents in the healthy control (HC) group were excluded if they had any psychopathology within the last two years, a lifetime diagnosis of a depressive disorder, a history of psychological treatment, or were taking medication for mental health issues or sleep disturbances. For adolescents diagnosed with depression, inclusion was permitted if the adolescent had a current primary diagnosis of Major Depressive Disorder (MDD) or dysthymia. Dysthymia is a persistent depressive disorder, with symptoms that are less severe but longer lasting than those of major depressive disorder. Adolescents in this group were excluded if they were using unstable doses of antidepressants, if there were safety concerns due to suicidal tendencies, severe self-harm, or if there were current comorbidities such as intellectual disability, psychotic disorder, eating disorder, substance use disorder, or autism spectrum disorder. The Kiddie-Schedule for Affective Disorders and Schizophrenia – Present and Lifetime (K-SADS-PL; Reichart et al., 2000) was used to assess both inclusion and exclusion criteria for the two groups with regards to mental health problems.

In total 80 HC and 35 adolescents with a depressive disorder participated in the RE-PAIR study. Two participants in each of these groups were missing both immune and epigenetic samples. Hence, 78 HC and 33 adolescents with depression were included in the current study (*N* = 111, mean age = 15.9 yrs, SD = 1.40, 68 % female) that either provided either immune or epigenetic data. The adolescents with a depression were less often of Dutch descent than the HC (33 % versus 9 %), and their parents reported lower average family income, but equal education levels compared to the control group. Descent and family income were not associated with any of the biological measures and hence not included in further analyses. Only 1 participant with a depression was currently on anti-depressant medication. The other adolescents with depression were on a waiting-list for treatment or had recently started with psychotherapy.

For the immune analyses we included all participants that were willing and able to give a saliva sample on the laboratory day (*n* = 108). This included 76 HCs (mean age = 16.0 yrs, SD = 1.25, 64 % female) and 32 adolescents with a depression (mean age = 15.6 yrs, SD = 1.55, 75 % female).

Due to financial constraints, we were only able to include 24 participants of each group in the epigenetic analyses. To this end, we selected 24 adolescents with a depression that were free of anti-depressant medication and largely free of any other medication and reported no to little physical health complaints on the laboratory day. We matched 24 HC to this group based on age and sex (HC: mean age = 15.7 yrs, SD = 1.43, 75 % female; adolescents with depression: mean age = 15.5 yrs, SD = 1.50, 75 % female), again including only participants that did not use any medication (except for 1 using allergy medication) and had no to little physical health complaints on the testing day. Three of these 48 participants had no immune data available but were still included in the relevant epigenetic age analyses.

### 2.2 Procedures

HC families were recruited through social media, advertisements, and flyers, while families with an adolescent experiencing depression were recruited through similar (online) advertisements and in collaboration with mental health care facilities in the Leiden area, the Netherlands. Families expressing interest in participation were given detailed information and underwent an initial screening via phone, which included a brief assessment of the inclusion and exclusion criteria. For families with an adolescent diagnosed with depression, a diagnostic appointment was scheduled, during which the adolescent was interviewed using the K-SADS-PL to further confirm eligibility. The K-SADS-PL assessment for the HC was completed at the end of the laboratory day.

Participation involved four components: completion of online questionnaires including the Childhood Trauma Questionnaire Short Form (CTQ-SF), a research day in the laboratory during which interaction tasks were performed between the parents and children and the biological assessments were done, 14 consecutive days of Ecological Momentary Assessment, and an fMRI scan session. Financial compensation was given depending on the components they completed, and travel expenses were reimbursed. A general debriefing procedure was included at the end of each test session and adolescents with depression also received written feedback based on their responses to several questionnaires completed before and during the research day. In the current study, we utilized data from the questionnaires and from the biological assessments.

The RE-PAIR study received approval in May 2018 from the Medical Ethics Committee of Leiden University Medical Centre (LUMC; NL62502.058.17) and was conducted in compliance with the Declaration of Helsinki and the Dutch Medical Research Involving Human Subjects Act (WMO). All participants provided informed consent, and for adolescents younger than 16 years, additional consent was obtained from parents with legal custody.

### 2.3 Measures

#### 2.3.1 Depression

The Kiddie-Schedule for Affective Disorders and Schizophrenia – Present and Lifetime (K-SADS-PL; Reichart et al., 2000) was used to establish a clinical diagnosis of depression, and was performed by trained psychologists or graduate students from the clinical psychology unit prior to the lab session in the depressed group, or by graduate students during the lab session (HC). Final diagnoses were discussed with a registered healthcare psychologist. Of the adolescents with depression, 80.0 % (*n* = 28) had current MDD and 20.0 % (*n* = 7) had current dysthymia. In addition, the patient health questionnaire (PHQ)-9 (Kroenke et al., 2001) was used to assess self-reported severity of depressive symptoms in the full sample. The PHQ-9 is based on the diagnostic criteria for MDD from the Diagnostic and Statistical Manual of Mental Disorders 5th Edition (American Psychiatric Association, 2022) and consists of 9 questions on a Likert scale from 0 to 3 that ask about the frequency of depression symptoms over the past two weeks.

#### 2.3.2 Childhood trauma

Experiences of childhood maltreatment were assessed via self-report with the Dutch version of the Childhood Trauma Questionnaire short form, CTQ-SF (Bernstein et al., 2003; Thombs et al., 2009), which includes the subscales: emotional abuse, emotional neglect, physical abuse, physical neglect, and sexual abuse. All subscales consist of five items, except the sexual abuse scale, which consists of four items due to a problem in the translation of an item (see Thombs et al., 2009), and were answered on a Likert scale from 1 (never true) to 5 (very often true). The additional minimization scale including 3 items was not included in the current analyses. The CTQ is a sensitive and reliable questionnaire that has been validated in a Dutch sample (Thombs et al., 2009). Internal consistency of the total scale in this sample was good with a Cronbach's alpha of 0.84. There were 2 outliers on the CTQ, hence data was standardized and these 2 values winsorized to 3 SD from the mean for analyses. For the exploratory follow-up analyses, emotional maltreatment was calculated by adding the emotional neglect and abuse scales (Cronbach's alpha = 0.88), and physical maltreatment was calculated by adding the physical neglect and abuse scales (Cronbach's alpha = 0.30). The low Cronbach's alpha of physical maltreatment was mostly due to a very low reliability of the physical neglect (0.04) versus the physical abuse scale (0.65), and hence only the physical abuse scale was included in the post-hoc analyses for emotional versus physical maltreatment.

#### 2.3.3 Immune measures

The immune markers were assessed from saliva collected via passive drool always at the same time during the morning of the laboratory day, shortly before lunch. Participants did not eat or drink anything in the hour before the sampling, although water was allowed up to 30 min before sampling. When possible, 2 ml of saliva was collected, and immediately stored at −20 °C until analyses. Analyses were performed at Danone Nutricia Research in Utrecht the Netherlands.

Interleukin (IL)-1β, IL-6, IL-8, and TNF-α were assessed using a 4-plex Luminex Discovery assay (R&D systems) according to the manufacturer's instructions. Intra- and inter-assay variation was assessed following the European Medicines Agency (EMA) guidelines for clinical trials (2011). Intra-assay variation was <15 % and inter-assay variation <20 %. Sensitivity of the different cytokines was 1.65 pg/ml (IL1-β), 0.41 pg/ml (IL-6), 0.35 pg/ml (IL-8) and 0.73 pg/ml (TNF-α). Saliva samples were analyzed undiluted and did not undergo any freeze-thaw cycles before the current analysis.

Secretory IgA [sIgA] was measured using an in-house Enzyme-linked Immunosorbent Assay (ELISA), fully validated for saliva and human milk samples (Dingess et al., 2021). Saliva samples were measured in a 100x and 500x dilution, and sensitivity of the assay was 5000 ng/ml. C-reactive protein (CRP) was measured using an ELISA kit (Salimetrics) according to the manufacturer's instructions, and sensitivity of the assay was 250 pg/ml. Intra- and inter-assay variation were <15 % for sIgA and CRP.

In seven participants (5 %) of the total sample no saliva was collected due to changes in protocol or insufficient saliva, and they were pairwise deleted from the analyses (*n* for immune analyses is 108). For IL-8, data was higher than expected and 73 data points (68 %) were extrapolated using the Flexmap 3D software xPONENT 4.2. For sIgA there was 1 participant with a value below detection limit, which was replaced by the detection limit divided by 2 (i.e., 2500 ng/ml). However, for CRP 58 values (54 %) were below detection limit, indicating that the ELISA kit was not sensitive enough for the current salivary analyses. To still explore potential group differences in CRP, the DNA methylation data was used to estimate CRP levels using a methylation-based signature of CRP levels (Conole et al., 2023; Suleri et al., 2024). Beta methylation values from 7 CpGs (of which 5 were present in the current dataset after quality control with the Meffil package in R) were multiplied by values derived from a large GWAS on CRP (Ligthart et al., 2016) to create a composite score, the CRP methylation profile (CRP-MP), which is reflective of basal CRP values.

Due to skewness of the data, all immune values were log-transformed before analyses. Furthermore, data was standardized and outliers were winsorized to 3 standard deviations (SD) from the mean to reduce the impact of outliers in the analyses (1 value for sIgA and 1 value for IL-6, from separate participants).

#### 2.3.4 Epigenetic aging

Buccal cells were collected with 2 cheek swabs (left and right) from which genomic DNA was extracted and bisulphite-converted before genome-wide DNA methylation was estimated using the Infinium EPIC 850k array. Quality control (QC) of the methylation data was performed using the Meffil package in R (Min et al., 2018), whereby probes were removed in case of a detection p-value >0.01 in more than 10 % of samples and/or with a bead number below three in more than 10 % of samples. Epigenetic age according to the PedBE (Mcewen et al., 2019) and the Horvath clock (Horvath and Raj, 2018) were estimated from Noob-method normalized beta methylation values using the Methylclock package in R (Pelegi-Siso et al., 2021). For the PedBE clock all 94 CpG loci were available, while for the Horvath clock 334 of the 353 CpG loci were available (95 %), as some CpGs did not pass the QC detection thresholds.

Separate linear regression models were used to regress chronological age on the epigenetic age estimates and the resulting residuals provided the measures of epigenetic age acceleration (EAA). Positive EAA values indicate a higher-than-expected biological age, whereas negative values indicate a lower-than-expected biological age, which would indicate epigenetic age *de*celeration. Cell type proportions were estimated with the Meffil package in R using the Houseman algorithm (Houseman et al., 2012) and buccal cell proportions were regressed onto the EAA values. The resulting residuals represent cell count-corrected EAA values that were used for the statistical analyses.

#### 2.3.5 Covariates

Covariates of interest were age, sex and body mass index (BMI; for its potential associations with inflammation and EAA (Etzel et al., 2022)). BMI was calculated based on measures of height and weight on the lab day with a mean of 21.2 (SD = 3.43). Due to 2 outliers in BMI (>30), data was standardized and then winsorized for these 2 cases for analyses. For the immune analyses, further possible covariates included self-report measures of medication use that may interfere with the inflammatory measures (e.g., anti-depressants, allergy medication, pain medication), smoking (at least once per week), measures of general health (e.g., cold or flu-like symptoms, allergies, iron deficiency), and of oral health (e.g. bleeding gums, mouth ulcers), which were dummy coded for analyses (present/not present). Lastly, while data collection of the HC was completed by the end of 2019, part of the depressed sample (66 %) was tested after the Covid lock-down procedures had come into effect. While data collection was temporarily paused, the study was continued after 6 months with more stringent rules regarding health. That is, no signs of illness or a cold, or a recent infection with Covid were allowed on the testing day. To assess the impact of these measures on the immune markers, we ran a post-hoc analysis in the depressed sample with a dummy coded variable representing before and after the start of the pandemic.

### 2.4 Analyses

Descriptive statistics of all measures were calculated, as well as correlations between the immune and epigenetic markers. To test whether the adolescents with depression differed from the HC on the immune markers IL-1β, IL-6, IL-8, TNF-α, and sIgA, a multivariate ANOVA (MANOVA) was performed with the 5 immune markers as multivariate dependent measures of inflammation, and group (adolescents with depression versus HC) as fixed predictor, followed by post-hoc univariate ANOVAs per immune marker in the case of a significant overall test. A separate ANOVA was performed on the CRP-MP measure. Next, to assess whether any differences between the groups on the immune markers could be attributed to experiences of childhood maltreatment, the CTQ was added as a continuous (covariate) variable to the MANOVA in a second step. Furthermore, in case of any significant correlations between the EAA and immune markers, we would also examine whether any group differences in the immune measures could be explained by EAA, by additionally including EAA as a continuous predictor in the MANOVA. Lastly, covariates that were associated with either group or any of the immune markers, were included in a last step to assess robustness of the findings.

Similarly, a MANOVA was performed with the PedBE and Horvath clocks as multivariate dependent measures of EAA, assessing differences between groups (adolescents with depression versus HC). Post-hoc univariate analyses per clock were again performed in the case of a significant overall test. Next, to assess whether any differences in EAA between the groups were due to experiences of childhood maltreatment, the CTQ was added to the MANOVA in a second step. Covariates that were associated with either group or the EAA measures were included in the last step to assess robustness of the findings.

Exploratory follow-up regression analyses were performed to examine first whether the severity of depression symptoms as measured with the PHQ-9 contributed to any of the group differences, and second whether childhood emotional or physical maltreatment (/abuse) better predicted any significant outcomes for childhood maltreatment. The above-mentioned analyses were performed in SPSS 29.0.2, using an alpha of 0.05 for the MANOVA omnibus analyses, preliminary and exploratory analyses. Univariate post-hoc tests for the immune measures were compared to a Bonferroni-adjusted alpha of 0.01 (5 tests), and to an alpha of 0.025 for EAA (2 tests). For the MANOVAs η_p_^2^ is presented as an effect size with 0.01, 0.06, and 0.14 representing small, medium and large effects (Richardson, 2011).

## 3 Results

Table 1 shows the descriptives statistics for the raw immune levels (before log transformation) and the cell count-corrected EAA and CRP-MP measures, as well as the CTQ scores and covariates, per group and for the full sample. As expected, the adolescents with depression scored significantly higher on the PHQ-9 than the HCs (*p* < .001) indicating more severe levels of depressive symptoms. Also, with regards to childhood maltreatment, adolescents with depression experienced more maltreatment during their childhood compared to the HC (*p* < .001), including both emotional maltreatment (*p* < .001) and physical abuse (*p* = .029). With regards to the covariates age, sex, BMI, (oral)health, smoking, and medication, there were no significant differences between the depressed group and HC (all *p*'s > 0.06).

**Table 1:** Descriptive statistics of the biological measures and possible covariates in the full sample, healthy control group, and group with depression.

	Full Sample			Healthy Control			Depression		
	n	Mean/n (%)	SD	n	Mean/n (%)	SD	n	Mean/n (%)	SD
sIgA (μg/ml)	108	194.16	118.14	76	180.74	107.78	32	226.02	136.30
IL-1β (pg/ml)	108	693.29	593.47	76	713.69	634.21	32	644.84	488.98
IL-6 (pg/ml)	108	14.98	43.50	76	17.14	51.48	32	9.84	8.95
IL-8 (pg/ml)	108	1036.78	477.79	76	1100.23	490.67	32	886.10^c^	414.91
TNF-α (pg/ml)	108	10.50	6.03	76	10.98	6.37	32	9.35	5.03
EAA (Horvath)	48	0.00	1.57	24	−0.13	1.33	24	0.13	1.80
EAA (PedBE)	48	0.00	1.06	24	0.03	0.98	24	−0.03	1.16
CRP-MP	48	0.00	0.99	24	−0.11	1.01	24	0.11	0.98
PHQ (severity)	111	9.29	7.74	78	4.85	2.96	33	19.79^b^	4.78
CTQ total	110	30.85	6.53	77	29.08	5.37	33	35.00^b^	7.18
CTQ emotional	110	15.89	5.63	77	14.39	4.74	33	19.39^b^	6.05
CTQ physical^a^	110	5.16	0.75	77	5.06	0.29	33	5.39^c^	1.27
BMI	111	21.22	3.46	78	20.78	2.65	33	22.25	4.77
Age	111	15.86	1.40	78	15.95	1.33	33	15.65	1.54
Sex (female)	111	75 (68 %)		78	50 (64 %)		33	25 (76 %)	
Medication use	111	35 (32 %)		78	21 (27 %)		33	14 (42 %)	
General health problems	111	33 (30 %)		78	22 (28 %)		33	11 (33 %)	
Oral health problems	111	22 (20 %)		78	16 (21 %)		33	6 (18 %)	
Smoking	111	16 (14 %)		78	11 (14 %)		33	5 (15 %)	

*Notes*: SD = standard deviation, sIgA = secretory immunoglobulin A, IL = interleukin, TNF = tumor necrosis factor, EAA = epigenetic age acceleration, CRP-MP = C-reactive protein - methylation profile, PHQ = patient health questionnaire (depressive symptoms), CTQ = childhood trauma questionnaire, BMI = body mass index.

a Physical abuse scale only.

b Group difference *p* < .01.

c Group difference *p* < .05.

The average epigenetic age according to the PedBE clock (mean age = 15.7 yrs, SD = 2.27) was higher than the Horvath clock (mean age = 13.7 yrs, SD = 2.72) and closer to chronological age (*n* = 48, mean age = 15.6 yrs, SD = 1.46). Correlations with chronological age were 0.70 (*p* < .001) for the PedBE clock and 0.64 (*p* < .001) for the Horvath clock. The EAA estimates of the PedBE and Horvath clocks showed a moderate correlation (*r* = 0.28, *p* = .047).

Table 2 and Supplemental Fig. 1 show Pearson correlation coefficients between all immune markers and epigenetic measures, and with the covariates (with the categorical variables dummy-coded to 0 and 1). The interleukins IL-1β, IL-6, IL-8 and TNF-α showed high correlations amongst themselves (*r*'s > 0.65, *p*'s < 0.001), and moderate correlations with sIgA (0.20 < *r*'s < 0.31, *p*'s < 0.03). No associations were found between these salivary immune markers and the CRP-MP score (*p*'s > 0.11). In contrast to expectations, no associations were found between any of the immune markers and either the PedBE or Horvath EAA measures (all *p*'s > 0.09). Females showed higher CRP-MP values, BMI was associated with higher sIgA levels, and oral health problems were related to higher IL-6 and IL-8 levels. Hence, in the multivariate analyses with the immune markers, gender, BMI and oral health problems were included as possible covariates. No relevant covariates were present for the EAA measures.

**Table 2:** Correlations between all biomarkers and possible covariates.

	IL-1β	IL-6	IL-8	TNF-α	sIgA	CRP-MP^d^	EAA (Horvath)^d^	EAA (PedBE)^#^
IL-1β		0.66^a^	0.80^a^	0.79^a^	0.30^b^	−0.24	−0.016	0.034
IL-6			0.71^a^	0.67^a^	0.21^c^	0.13	−0.17	−0.041
IL-8				0.80^a^	0.29^b^	0.036	0	0.11
TNF-α					0.31^b^	0.22	−0.023	0.25
sIgA						−0.086	0.25	−0.081
CRP-MP^d^							0.23	0.20
EAA (Horvath)^d^								0.29^c^
Age	0.008	0.11	0.053	0.01	0.15	0.26	0	0
Sex (female)	−0.077	−0.14	−0.089	−0.081	−0.18	0.30^c^	−0.014	0.03
BMI	0.057	−0.016	−0.028	−0.026	0.24^c^	−0.036	−0.029	−0.054
Medication use	−0.049	−0.058	−0.15	−0.099	0.089	−0.093	0.083	−0.091
Health problems	−0.007	−0.090	−0.085	0.048	0.054	0.077	−0.001	−0.066
Oral health problems	0.15	0.20^c^	0.22^c^	0.19	0.018	0.16	0.065	0.028
Smoking	0.055	−0.081	−0.025	0.027	−0.17	0.001	−0.16	0.10

*Notes*: IL = interleukin, TNF = tumor necrosis factor, sIgA = secretory immunoglobulin A, CRP-MP = C-reactive protein - methylation profile, EAA = epigenetic age acceleration, BMI = body mass index. ^a^ Physical abuse scale only.

a *p* < .001.

b *p* < .01.

c *p* < .05.

d cell count corrected.

### 3.1 Depression, maltreatment, and inflammation

We performed the first MANOVA with the immune markers IL-1β, IL-6, IL-8, TNF-α, and sIgA as measures of the dependent variable inflammation, and group as a fixed factor. The omnibus test showed that group was significantly associated with the immune markers (*F* (5, 102) = 3.245, *p* = .009, η_p_^2^ = 0.137). Univariate post-hoc tests showed that this effect was mostly driven by IL8, which was the only marker showing a nominally significant association with group (*F* (1, 106) = 5.047, *p* = .027, η_p_^2^ = 0.045), with a small to medium effect size. However, this finding did not survive correction for multiple testing (*p*-value >0.01). The omnibus effect of group remained significant when controlling for sex, BMI and oral health as covariates (*F* (5, 99) = 2.847, *p* = .019, η_p_^2^ = 0.126), as well as the uncorrected univariate effect of IL-8 (*F* (1, 103) = 4.694, *p* = .033, η_p_^2^ = 0.044, see Supplemental Table 1 for the full model. Even though the univariate tests did not survive multiple-testing correction, to be able to interpret the omnibus effect we examined the direction of the effect in IL-8. In contrast to expectations, levels of IL8 were lower in the group with depression versus the HC (depressed group mean_raw_ = 886 pg/ml, SD = 415 pg/ml; HC mean_raw_ = 1037 pg/ml, SD = 491 pg/ml), see Fig. 1.

**Fig. 1:**
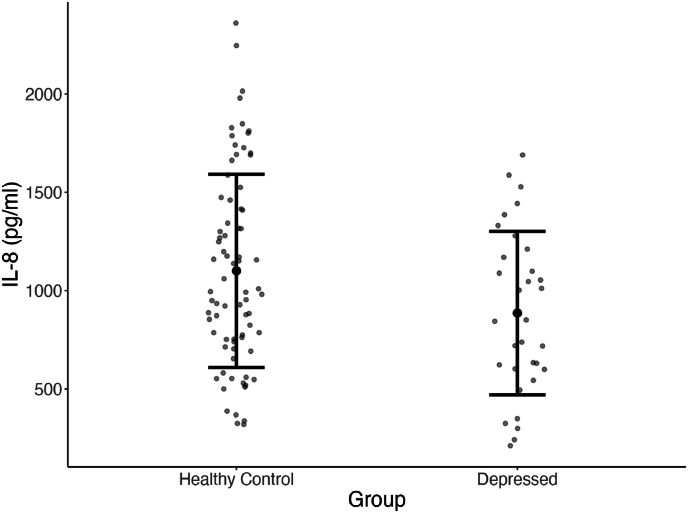
Raw levels of IL-8 (pg/ml) per group. Error bars show 1 standard deviation from the mean. The group difference of the log-transformed values of IL-8 was only nominally significant (*p* = .03).

CTQ was entered in a next block, but in contrast to expectations it did not show any associations with the immune markers (*F* (5, 100) = 0.260, *p* = .934, η_p_^2^ = 0.013). As the preliminary analyses showed that EAA was not associated with any of the immune measures, we did not perform the planned MANOVA with EAA as an additional predictor in the model.

Next, we included PHQ-9 scores as a predictor in the MANOVA instead of group, to explore whether the effects of group were driven by severity of the symptoms. While the omnibus effect of PHQ-9 was significant (*F* (5, 102) = 2.479, *p* = .037, η_p_^2^ = 0.108), it failed to reach significance for any of the univariate tests (*p's* > 0.06). Lastly, for the CRP-MP measure, a univariate ANOVA was performed repeating the above steps. However, no significant associations were found with group, the CTQ or the PHQ-9 (all *p*'s > 0.43).

Within the depressed group we explored whether there were any differences in the immune markers that were due to the start of the pandemic. We performed another MANOVA with the 5 immune markers as dependent variables and pre-post start of the Covid pandemic as a predictor, as well as an ANOVA with the CRP-MPS. The analyses showed no differences between pre- and post-Covid on the overall test (*F* (5, 26) = 1.501, *p* = .224, η_p_^2^ = 0.224), or any of the univariate post-hoc tests (all *p*'s > 0.10). For the CRP-MP there was a trend towards lower levels after the lockdown, but this did not reach significance (*F* (1,22) = 3.942, *p* = .06, η_p_^2^ = 0.152).

In sum, depression status was associated with differences in the immune system, possibly driven by lower levels of IL-8, which was not explained by experiences of childhood maltreatment or EAA.

### 3.2 Depression, maltreatment and epigenetic aging

We performed a MANOVA with the PedBE and Horvath clocks as measures of the dependent variable EAA, and group as a fixed factor. The omnibus test showed that in contrast to expectations, group was not significantly associated with EAA (*F* (2, 45) = 0.245, *p* = .784, η_p_^2^ = 0.011).

**Fig. 2:**
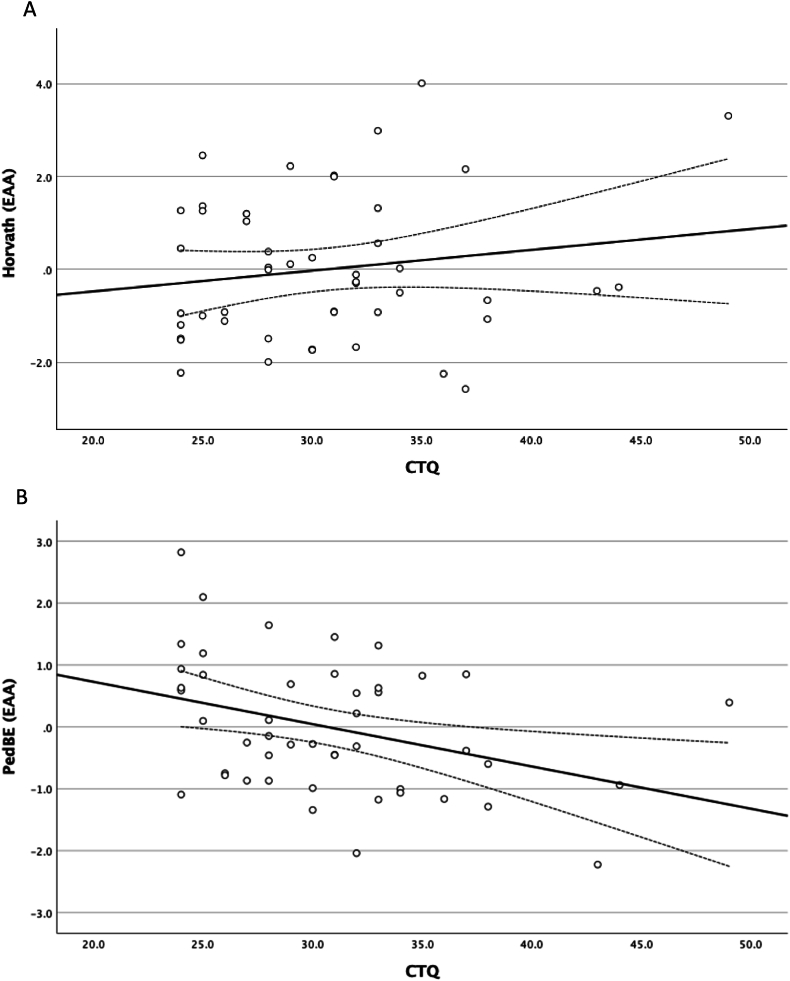
Associations between childhood maltreatment (CTQ) and epigenetic age acceleration (EAA) with the Horvath (A) and PedBE (B) clocks.

CTQ was entered in the next step. The omnibus test showed that the CTQ was significantly associated with EAA (*F* (2, 44) = 6.580, *p* = .003, η_p_^2^ = 0.230). Univariate post-hoc tests showed that this effect was driven by the PedBE clock, which was the only marker showing a significant association (*F* (1, 45) = 8.547, *p* = .005, η_p_^2^ = 0.160), also after correction for multiple testing (*p* < .025). In contrast to expectations though, higher levels of maltreatment, as indicated by the CTQ, were associated with lower levels of EAA assessed with the PedBE clock (beta = −0.363, *p* = .011, *R*^2^ = 0.13), which indicates that more experiences of childhood maltreatment were related to epigenetic age *deceleration* instead of acceleration. See Fig. 2A for the (non-significant) association with the Horvath clock clock (*F* (1, 45) = 0.869, *p* = .36, η_p_^2^ = 0.019), and Fig. 2B for the significant association with the PedBE clock.

This association seemed to be driven by emotional maltreatment (beta = −0.345, *p* = .018, *R*^2^ = 0.13) rather than physical abuse (beta = −0.005, *p* = .9713, *R*^2^ < 0.001), when examined together as predictors in a regression model. To further explore whether it was experiences of abuse versus neglect, we included the subscales emotional abuse, physical abuse and emotional neglect in one analysis, showing emotional abuse to be the only significant predictor (beta = −0.391, *p* = .039). Lastly, sensitivity analyses in which EAA was not corrected for buccal cells count did not lead to any different conclusions.

## 4 Discussion

In this study, we first examined the extent to which adolescent depression is associated with higher levels inflammation as indexed by salivary markers, and whether this association can be attributed to more experiences of childhood maltreatment. Second, we examined whether epigenetic age acceleration plays a role in any of these associations.

We found depression to be associated with the immune markers. However, in contrast to expectations, there was no indication of inflammation, and the only marker showing a univariate association with depression was IL-8, with *lower* levels being associated with depression. This association did not survive correction for multiple testing and hence these results must be seen as preliminary and carefully interpreted. Furthermore, the association of depression with the immune markers could not be explained by experiences of childhood maltreatment. Secondly, no associations were found between depression and epigenetic aging, nor between any of the immune markers and measures of epigenetic aging. Lastly, experiences of childhood maltreatment, in specific emotional maltreatment, were reversely associated with epigenetic age acceleration, i.e. with epigenetic age *deceleration*, indicating lower-than-expected epigenetic age. This association was only found for the PedBE clock, not the Horvath clock.

The finding of a potential suppression of the immune system rather than signs of heightened inflammation in the adolescents with a depressive disorder is not in line with the findings of chronic inflammation in adults with depression (Haapakoski et al., 2015; Osimo et al., 2020). However, the finding of heightened inflammation in adolescents and children with depression is inconsistent in the literature (Colasanto et al., 2020; D'Acunto et al., 2019; Toenders et al., 2021), and our findings tentatively add to the negative findings of inflammation in this age group. We only found suggestive evidence for lower levels of IL-8. When we look specifically into the literature for this immune marker, we see that lower levels of IL-8 have previously been associated with higher severity levels of depression in adult females (Kruse et al., 2021), more depressive symptoms over time in adolescent males (Moriarity et al., 2019), and with first episode or drug-free major depressive disorder in adulthood (Çakici et al., 2020; Zhu et al., 2022). In this regard, our largely medication-free and young group of adolescents matches this latter adult group. Possibly, lower levels of IL-8 are an early indicator of immune imbalances in the early stages of depression, with inflammation only showing up in later stages of depression or after longer exposure durations. Adolescents are still undergoing neurobiological and immunological development, and early immune imbalances, either lowered or heightened, may impact further brain development. A recent study has shown that reduced IL-8 levels might reflect abnormal activity of brain microglia and astrocytes (Zhu et al., 2022), and hence might be implicated in the development of depression. Whether IL-8 is of specific relevance in adolescence and first-time depression onset will be an avenue of further investigation.

Interestingly, we did not find any associations between the immune markers and experiences of childhood maltreatment, even though some theories pose that these stressful early life experience may cause the immune imbalances seen in depression (Danese and Lewis, 2016; Slavich and Irwin, 2014). Our group experienced relatively mild levels of childhood maltreatment though (82 % low emotional abuse, 66 % low emotional neglect, 99 % low physical abuse physical abuse and 92 % low physical neglect). This may be because families came to the lab together, which may have biased our sample to include relatively supportive parents. Other life stressors (e.g. bullying, family conflict) may hence have been more relevant in the development of possible immune dysregulations. On the other hand, it may also be that the association between childhood maltreatment and the immune system does not show up till much later in life, as a recent longitudinal study with a composite inflammatory biomarker indicates (Crick et al., 2022).

With regards to epigenetic age acceleration, no associations with depression were found, which contrasts with previous findings in adult depression and some findings in children with higher levels of internalizing and (non-clinical) depressive symptoms (Harvanek et al., 2023; Perret et al., 2023; Shindo et al., 2023; Suarez et al., 2018b; Tollenaar et al., 2019). Some studies indicate that depressive symptoms interact with childhood maltreatment in predicting epigenetic age acceleration (Dammering et al., 2021; Zhang et al., 2023). Our depressive sample was too small to study moderation, and this would be of interest for future studies in adolescents. Although, we did find associations of epigenetic aging with experiences of childhood maltreatment in the full sample. However, this association was in the opposite direction of our expectations, as we found maltreatment to predict epigenetic age deceleration, while most studies show associations between early life stress and accelerated levels of epigenetic aging in children and adolescents (Copeland et al., 2022; Jovanovic et al., 2017; Marini et al., 2020).

Interestingly though, epigenetic age deceleration has also been shown in different clinical groups including individuals with post-traumatic stress disorder (Boks et al., 2015; Verhoeven et al., 2018), children with externalizing problem behavior who experienced harsh parenting (Creasey et al., 2024), and children from residential care (Meier et al., 2024). Furthermore, the type of experienced stressor may relate to the type of epigenetic age deviation with stronger indications for acceleration after experiences of threat versus neglect (Hamlat et al., 2021; Hogan et al., 2024; Rampersaud et al., 2022; Sumner et al., 2019). These earlier findings suggest that deviations in either direction (acceleration or deceleration) can be indicative of problems in healthy development. Notably, however, in our sample the association with epigenetic deceleration was driven by emotional abuse and not neglect. Our unmedicated, physically healthy, adolescent sample may represent a specific group in which epigenetic age deceleration represents an adaptive response or a delay in development due to experienced early life emotional stressors. Whether such developmental delays in adolescence lead to compensatory epigenetic age accelerations in later life, or after longer trauma exposures, is yet unknown. And while epigenetic age acceleration in adulthood has been associated with disease and early mortality, the impact of epigenetic age deviations in childhood and adolescence for later mental and physical health remains largely unknown. In this regard, longitudinal studies (e.g. Mastrotheodoros et al., 2023) are highly recommended, in which deviations in epigenetic age from a young age on are studied in relation to the development of both physical and mental health problems into adulthood. Furthermore, our study shows that the use of different epigenetic clocks may lead to different outcomes. The PedBE clock was specifically trained on pediatric data and was able to capture the impact of childhood maltreatment in our sample. Future studies should carefully select the most relevant clock for their research question, collection method (saliva or blood), and age range, especially in longitudinal designs.

Strengths of our study include the use of a large range of immune markers and the comparison with DNA based methylation scores. While our sample was not as large as some cohort studies in this area, in those studies levels of depressive or internalizing symptoms are usually relatively low and may not cover symptoms in the clinical range. We were able to include a group of adolescents with a clinically diagnosed depressive disorder and carefully match them to a healthy control group in a case-control design. However, for smaller effects sizes we were likely underpowered, and our sample size did not allow us to look further into subgroups of depressive symptoms, the role of comorbidities, duration or chronicity. Also, the role of individual differences like medication use and BMI can only be studied in larger, more heterogeneous samples. To minimize differences in physical health complaints and medication use, we furthermore selected participants with only few physical health complaints and no (antidepressant) medication for the epigenetic analyses. While this gives us a homogenous group comparison, it may limit generalizability to the broader clinical population of adolescents with depression.

Drawbacks are furthermore that we could not do sex specific analyses. While sex may play an important role in the relation between depression and inflammation (Toenders et al., 2021), our sample was underpowered to examine interactions with sex, with only 7 boys (20 %) in the depressed sample, even though this is representative of the sex distribution of depression in the population. Also, the age at which childhood maltreatment was experienced was not assessed, and we could therefore not examine whether there are different sensitive periods for early life stress to impact the immune system and epigenetic aging. Furthermore, pubertal development was not assessed and could have impacted the association between stress and the immune system (Holder and Blaustein, 2014), as well as the assessments of epigenetic age (Sumner et al., 2023b).

With regards to the number of tests in our sample, we must be careful in the interpretation of the outcome on the IL-8 measure, as the post-hoc univariate test did not survive more strict statistical correction. Also, the measurement of immune markers in saliva has received considerable debate (Engeland et al., 2019; Riis et al., 2014; Shields et al., 2019), as it is not fully clear to which extent salivary assessments reflect more systemic levels of immune functioning. We do show associations with oral health, which we controlled for in the analysis, but also find associations with a more systemic health outcome, i.e., BMI, indicating saliva may also pick up potential systemic immune dysregulations. However, the assay we used for salivary CRP analyses was not sensitive enough. While we used a methylation-based proxy (CRP-MP) as an alternative, this measure did not correlate with any of the other immune markers and clinical measures. Hence, it is unclear to what extent this proxy represents systemic CRP levels, which reduces the conclusions we can make about changes in inflammation. Lastly, the physical neglect scale of the CTQ did not reach an adequate level of reliability in our sample. This may be due to the low occurrence, but also because of the relatively young sample (Hagborg et al., 2022). Our results on the impact of neglect are therefore limited to the emotional neglect scale.

In sum, our preliminary findings that adolescent depression is associated with reduced rather than elevated immune markers and that childhood maltreatment is associated with epigenetic age *de*celeration, contrast with previous literature in adults, indicating that epigenetic age *ac*celeration and inflammation are not unequivocal indicators of depression and childhood trauma in adolescents. However, considering our small, largely unmedicated sample, the results should be interpreted with caution, and require replication in larger and more heterogenous samples. Future studies should investigate under which circumstances increased or decreased levels of these biological markers indicate vulnerability for health-related outcomes. Further investigation of the interplay between childhood maltreatment, immune dysregulation, and adolescent depression is needed to inform targeted interventions and preventive strategies aimed at reducing both the psychological and physical burden of depression. There is some evidence that the impact of early life adversity on biological aging processes may be mitigated by early intervention (Brody et al., 2016; Creasey et al., 2024; Sullivan et al., 2023), which makes it even more pressing to understand the underlying mechanisms and individual differences in these processes.

## CRediT authorship contribution statement

**Marieke S. Tollenaar:** Conceptualization, Data curation, Formal analysis, Funding acquisition, Supervision, Visualization, Writing – original draft. **Nicole Creasey:** Data curation, Formal analysis, Writing – review & editing. **Mirjam C.M. Wever:** Investigation, Methodology, Project administration, Writing – review & editing. **Karen Knipping:** Conceptualization, Funding acquisition, Investigation, Methodology, Writing – review & editing. **Johan Garssen:** Methodology, Writing – review & editing. **Lisanne E.A.M. van Houtum:** Investigation, Methodology, Project administration, Writing – review & editing. **Wilma G.M. Wentholt:** Investigation, Methodology, Project administration, Writing – review & editing. **Loes Janssen:** Investigation, Methodology, Project administration, Writing – review & editing. **Elad Lax:** Conceptualization, Funding acquisition, Writing – review & editing. **Bernet M. Elzinga:** Conceptualization, Funding acquisition, Supervision, Writing – review & editing.

## Declaration of Generative AI and AI-assisted technologies in the writing process

During the preparation of this work, the authors used ChatGPT to help identify and correct potential grammatical errors or awkward phrasing in the Introduction and Discussion sections, as the first author's native language is Dutch. After using this tool, the authors reviewed and edited the content as needed and take full responsibility for the content of the publication.

## Funding

This study was supported by a personal research grant from the Netherlands Organization for Scientific Research awarded to BME. (VICI; Unravelling the Impact of Emotional Maltreatment on the Developing Brain 453–15–006); Ariel University; and Stichting S.K.A.I.

## Declaration of competing interest

JG is partly employee of Danone Nutricia Research. The authors declare that they have no known competing financial interests or personal relationships that could have appeared to influence the work reported in this paper.

## Figures and Tables

**figs1:**
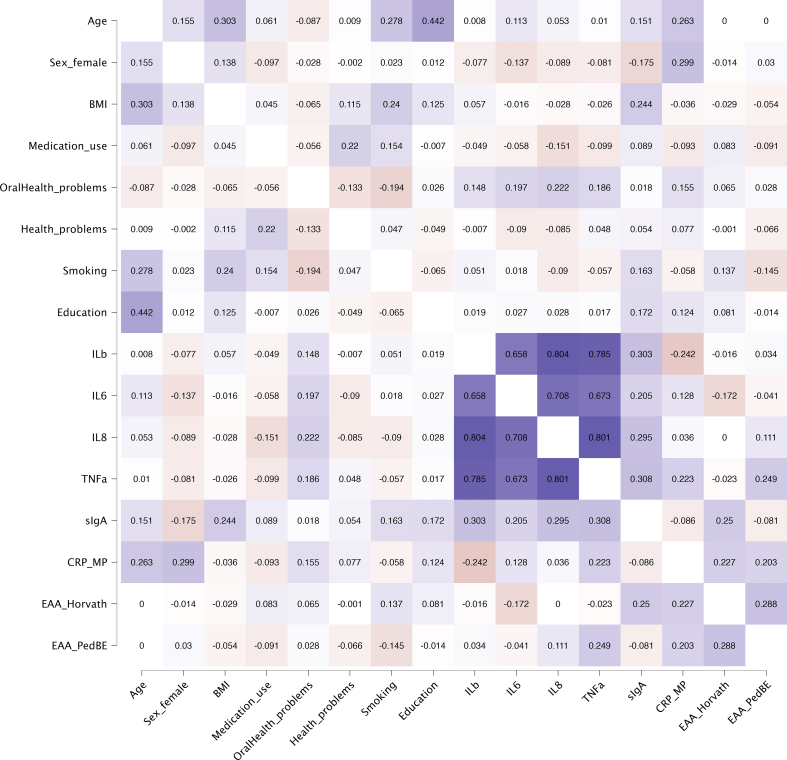
Heatmap of correlations between all biomarkers and possible covariates.

## Data Availability

Data will be made available on request.
